# Impact of the COVID-19 Pandemic on the Loading and Quality of an Emergency Department in Taiwan: Enlightenment from a Low-Risk Country in a Public Health Crisis

**DOI:** 10.3390/jcm10061150

**Published:** 2021-03-10

**Authors:** Jamie Yu-Hsuan Chen, Feng-Yee Chang, Chin-Sheng Lin, Chih-Hung Wang, Shih-Hung Tsai, Chia-Cheng Lee, Sy-Jou Chen

**Affiliations:** 1 Medical Informatics Office, Tri-Service General Hospital, National Defense Medical Center, Taipei City 11490, Taiwan; jamiechen@mail.ndmctsgh.edu.tw; 2 School of Public Health, National Defense Medical Center, Taipei City 11490, Taiwan; 3 Division of Infectious Diseases and Tropical Medicine, Department of Medicine, Tri-Service General Hospital, National Defense Medical Center, Taipei City 11490, Taiwan; fychang@mail.ndmctsgh.edu.tw; 4 Division of Cardiology, Department of Medicine, Tri-Service General Hospital, National Defense Medical Center, Taipei City 11490, Taiwan; littlelincs@gmail.com; 5 Department of Otolaryngology-Head and Neck Surgery, Tri-Service General Hospital, National Defense Medical Center, Taipei City 11490, Taiwan; chw@ms3.hinet.net; 6 Graduate Institute of Medical Sciences, National Defense Medical Center, Taipei City 11490, Taiwan; 7 Department of Emergency Medicine, Tri-Service General Hospital, National Defense Medical Center, Taipei City 11490, Taiwan; doc50024@mail.ndmctsgh.edu.tw; 8 Division of Colorectal Surgery, Department of Surgery, Tri-Service General Hospital, National Defense Medical Center, Taipei City 11490, Taiwan; 9 Graduate Institute of Injury Prevention and Control, College of Public Health and Nutrition, Taipei Medical University, Taipei City 11490, Taiwan

**Keywords:** chest pain, COVID-19, emergency departments, length of stay, prognosis

## Abstract

The impact of the coronavirus disease 2019 (COVID-19) pandemic on health-care quality in the emergency department (ED) in countries with a low risk is unclear. This study aimed to explore the effects of the COVID-19 pandemic on ED loading, quality of care, and patient prognosis. Data were retrospectively collected from 1 January 2018 to 30 September 2020 at the ED of Tri-service general hospital. Analyses included day-based ED loading, quality of care, and patient prognosis. Data on triage assessment, physiological states, disease history, and results of laboratory tests were collected and analyzed. The number of daily visits significantly decreased after the pandemic, leading to a reduction in the time to examination. Admitted patients benefitted from the pandemic with a reduction of 0.80 h in the length of stay in the ED, faster discharge without death, and reduced re-admission. However, non-admitted visits with chest pain increased the risk of mortality after the pandemic. In conclusion, the COVID-19 pandemic led to a significant reduction in low-acuity ED visits and improved prognoses for hospitalized patients. However, clinicians should be alert about patients with chest pain due to their increased risk of mortality in subsequent admission.

## 1. Introduction

The coronavirus disease 2019 (COVID-19) has spread to multiple countries after the initial outbreak in Wuhan, China [1]. Currently, this novel virus has caused more than 81 million confirmed cases and 1.8 million deaths in 2020. This unprecedented pandemic has changed daily life and medical service usage. Studies have reported decreased health-care services usage, particularly for non-COVID-19 visits to the emergency department (ED) [2] due to public panic and the fear of contacting suspected COVID-19 patients [3]. Delays in symptom onset to hospital arrival time are also common. The median stroke onset-to-door time was approximately 1 h longer after the start of the COVID-19 pandemic [4], and a significant increase in out-of-hospital cardiac arrest (OHCA) was reported [5,6]. These delayed visits may lead to worse prognoses compared to before the COVID-19 pandemic [7].

Preventive measures are necessary to mitigate spread of the COVID-19 pandemic, particularly in the ED for frontline medical providers during this crisis. Implementation of ED staff safety protocols is also of utmost importance, with provisions and guidelines for personal protective equipment and team segregation to ensure staff safety [8]. The fever screening station takes precedence during the COVID-19 pandemic [9]. However, some studies have reported worse medical quality due to increased workload and chaotic traffic flow. Clinicians should wear full personal protective equipment to perform resuscitation, which may cause a delay that could potentially lead to poor outcomes [10]. Longer door-to-balloon time for primary percutaneous coronary intervention was also noted [11]. These non-COVID-19 conditions have been adversely influenced during the COVID-19 pandemic.

Overcrowding is the most serious problem in EDs worldwide [12]. The COVID-19 pandemic has caused decreased medical utilization [13,14,15,16], which also influences non-COVID-19 visits to the ED [17]. Since EDs have taken major responsibility for fever screening during the COVID-19 pandemic, the impact on medical quality warrants investigation. Length of stay in the ED is considered one of the most important quality indicators. A prolonged length of stay in the ED is associated with a reduced quality of care and worse outcomes [18,19,20,21]. Time to examinations are also critical because subsequent treatments are highly dependent on their results [22]. For prognostic analysis, discharge time is the most important indicator for general prognosis in admitted patients [23]. For outpatient visits, return to the ED is associated with adverse events and increased costs [24]. Moreover, mortality and re-admission are important indicators for measuring ED quality [18,19]. Most studies have reported substantial impacts from the COVID-19 pandemic on non-COVID-19 cases [3,25]. To the best of our knowledge, there has been no ED quality assessment since the COVID-19 pandemic.

Taiwan quickly mobilized and instituted specific approaches to protect its citizens from the COVID-19 pandemic [26]. However, there are limited studies regarding the effects of preventive measures or inevitable public panic on ED quality in low-risk countries since the COVID-19 pandemic. This study aimed to explore changes in ED loading, care quality in the ED, and patient prognosis after the start of the COVID-19 pandemic.

## 2. Materials and Methods

### 2.1. Study Design and Settings

This study was approved by the institutional review board of Tri-Service General Hospital (IRB NO. A202005159). This study was performed in the ED of an academic level 1 medical center in Taipei, which has approximately 1700 beds and an approximately 100,000 annual ED volume. We retrospectively collected data from 1 January 2018 to 30 September 2020, which included the time before and after the start of the COVID-19 pandemic. The most drastic change in the ED after the start of the pandemic involved setting up fever screening stations for separation of cases with COVID-19 risk, which started on 6 February 2020 and was guided by central preventive policy. Therefore, we defined 6 February 2020 as the date to distinguish before and after the start of the COVID-19 pandemic.

### 2.2. Data Source and Visit-Based Variable Measurements

All visits to the ED received initial assessment at the triage station. Nurses perform a focus assessment and categorize patients from level 1 (life threatening) to level 5 (non-urgent, outpatient) [27]. They measure and record the physiological state, including gender, age, body mass index (BMI), systolic blood pressure (SBP), diastolic blood pressure (DBP), temperature, pulse, and breathing. Certain common tags, including coma, chest pain, abdominal pain, trauma, and fever, were annotated according to the corresponding chief complaints and physiological assessment. We excluded level 5 visits from analysis because the majority of them were for certification of a negative COVID-19 test. Moreover, 28 confirmed COVID-19 cases were also excluded [28]. These data were obtained from the electronic database of the triage system.

Disease histories were obtained from the electronic medical records based on corresponding International Classification of Diseases, Tenth Revision (ICD-10), which included diabetes mellitus (DM, ICD-10 codes E11.x), hypertension (HTN, ICD-10 codes I10.x to I16.x), chronic kidney disease (CKD, ICD-10 codes N18.x), stroke (ICD-10 codes I60.x to I63.x), coronary artery disease (CAD, ICD-10 codes I20.x to I25.x), and hyperlipidemia (HLD, ICD-10 codes E78.x). Patients received corresponding examinations, including X-ray examination, electrocardiogram, and laboratory tests, after physician assessment. Time to X-ray examination, time to electrocardiogram, and time to laboratory tests were calculated from time at the triage station to first time of each examination being recorded. We collected the results of laboratory tests of patients, including creatinine (Cr), blood urea nitrogen (BUN), aspartate aminotransferase (AST), alanine aminotransferase (ALT), glucose (GLU), troponin I (TnI), C-reactive protein (CRP), white blood cell count (WBC), and platelet (PLT). All visits were divided into admitted and non-admitted groups. Length of stay was measured from the time at the triage station to the time at admission for the admitted visits or at discharge for non-admitted visits.

For admitted visits, we analyzed the odds of discharge by length of inpatient stay without death, which was calculated from the admission date to the date of discharge. Cases of in-hospital death were censored data in this analysis. Moreover, we analyzed events of re-admission within 30 days, which only used living discharged cases and followed up from date of discharge. Death in 30 days included in-hospital death starting from the admission date. For non-admitted visits, the rates of ED revisit in 3 days, admission in 30 days, and death in 30 days were analyzed. The follow-up time of these three outcomes started from the date of ED discharge in non-admitted visits. All prognoses were collected from the ED and inpatient electronic medical records.

### 2.3. ED Loading Parameters

Because treatment priority at the ED is closely related to disease severity and the initial assessment at triage, the wait time of each visit was highly dependent on the number of patients with different severities at the same time. We calculated the corresponding numbers of patients staying in the ED according to the time of each visit at triage to represent ED loading. Moreover, each visit was categorized into corresponding holiday, weekday, or daily rotating shift (07:30–19:30 and 19:30–07:30), according to the time the patient presented to triage.

### 2.4. Statistical Analysis

All statistical analyses were conducted in R v3.4.4, and the significance level was set as *p* < 0.05. We first conducted day-based analysis to explore changes due to the COVID-19 pandemic. Daily values were processed as the number of patients in different tags annotated by triage station. The line chart demonstrates the means of daily values from month to month, and Poison regression with incidence rate ratios (IRR) and corresponding 95% confidence intervals (95% CI) were used to quantify ED loading changes after the start of the COVID-19 pandemic. The adjusted variables included the day-based variables.

Visit-based analyses were used to evaluate changes in ED quality after the start of the COVID-19 pandemic. Baseline characteristics and ED loading parameters are presented as the means and standard deviations, numbers of patients, or percentages, where appropriates. We compared differences before and after the start of the COVID-19 pandemic using Student’s *t*-test or Chi-squared test, as appropriate. Univariable and multivariable linear regression with difference (beta) and 95% CI were used to evaluate changes in length of stay after the start of the COVID-19 pandemic, which were conducted in all subgroups of triage tag. Adjusted variables included two stages. The first stage was to evaluate the impact of the COVID-19 pandemic after adjusting for the severity of patients, which included all variables of triage assessment, physiological state, and disease histories. Laboratory tests were excluded from adjustment due to incomplete measurements. The second stage was to additionally adjust ED loading parameters and time information to simulate the independent changes from the COVID-19 pandemic under the same situation. Similar analyses were conducted on time to X-ray examination, time to electrocardiogram, and time to laboratory test.

The final analysis of the study was to follow up on the prognoses, which stratified all visits into admitted and non-admitted due to different prognoses. The Kaplan–Meier curve was used to determine differences before and after the start of the COVID-19 pandemic. We also used univariable and multivariable Cox proportional hazard model to quantify changes before and after the start of the COVID-19 pandemic. The hazard ratios (HR) and 95% CIs were used for comparison. The adjusted variables included two stages, the same as the analysis for length of stay. The first stage was to adjust for the severity of patients, and the second stage included additional ED loading parameters.

## 3. Results

**Figure 1:**
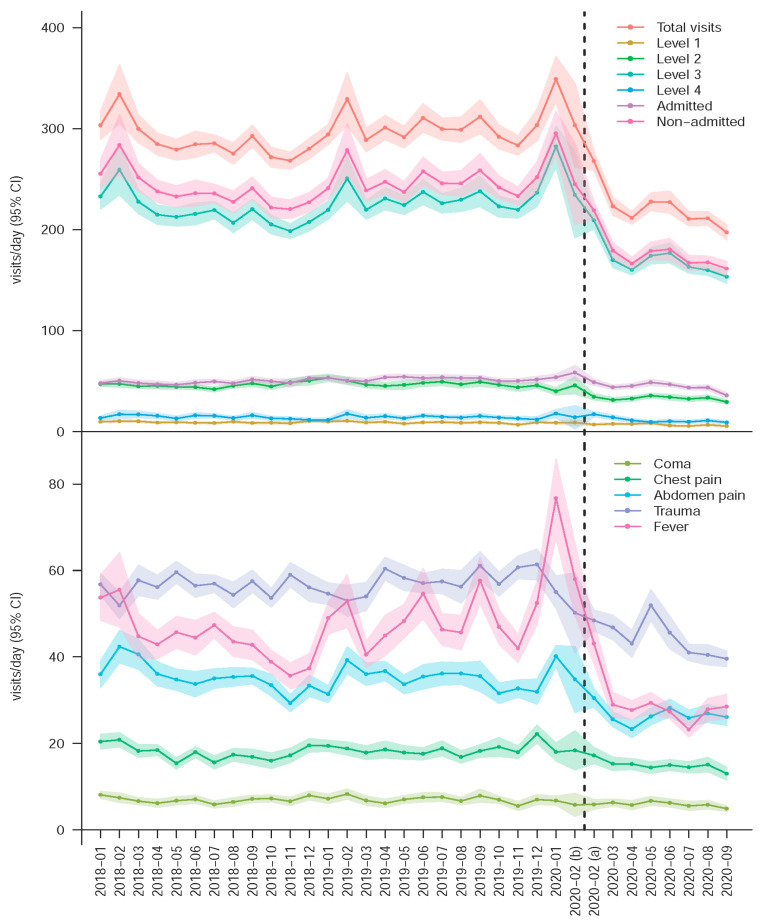
Daily visits to the emergency department (ED) by acuity and disease characteristics. The solid lines are the means and the areas are the 95% conference intervals. The dotted line is the divide between before and after the start of the COVID-19 pandemic.

Figure 1 shows monthly ED daily visits for different triage levels and diseases. The average daily visits decreased significantly after the start of the COVID-19 pandemic, which was presented for all subgroups. This apparent change can be attributed to the COVID-19 pandemic because the number of daily visits remained stable before the COVID-19 pandemic. Therefore, we combined all visits to explore the impact of the COVID-19 pandemic on ED loading. Table 1 shows a reduction of 25.5% in the number of total visits after the start of the COVID-19 pandemic [IRR: 0.745 (95% CI: 0.738–0.752)], with a greater drop in non-admitted visits [IRR: 0.718 (95% CI: 0.711–0.726)] compared to the number of the admitted visits [IRR: 0.875 (95% CI: 0.857–0.894)]. The number of visits with fever exhibited the greatest reduction [IRR: 0.612 (95% CI: 0.596–0.628)], particularly in the non-admitted visits [IRR: 0.537 (95% CI: 0.520–0.554)]. Interestingly, the number of admitted visits with chest pain was the only unchanged subgroup [IRR: 0.934 (95% CI: 0.845–1.033)], except for triage level 4 with very few numbers. The multivariable analysis demonstrated the independent effect of the COVID-19 pandemic on ED visits with similar results.

Table 2 shows the visit-based baseline characteristics before and after the start of the COVID-19 pandemic. After the start of COVID-19, patients have greater tendency to be classified as triage levels 3 and 4, comprising patients who were older, heavier, and more disease histories. Visits with coma, chest pain, abdominal pain, and trauma were increased as opposed to fever, which showed a significant reduction in both admitted and non-admitted visits. Although laboratory tests and vital signs revealed a significant difference after the start of the COVID-19 pandemic, the variations seem not to have clinical importance. Table 3 shows comparisons of visit-based ED loading parameters. The number of visits in all triage levels significantly decreased after the start of the COVID-19 pandemic, indicating a significant loading reduction in non-COVID-19 visits. The proportion of visits significantly decreased on holidays and during the night shift, particularly for non-admitted visits.

**Figure 2:**
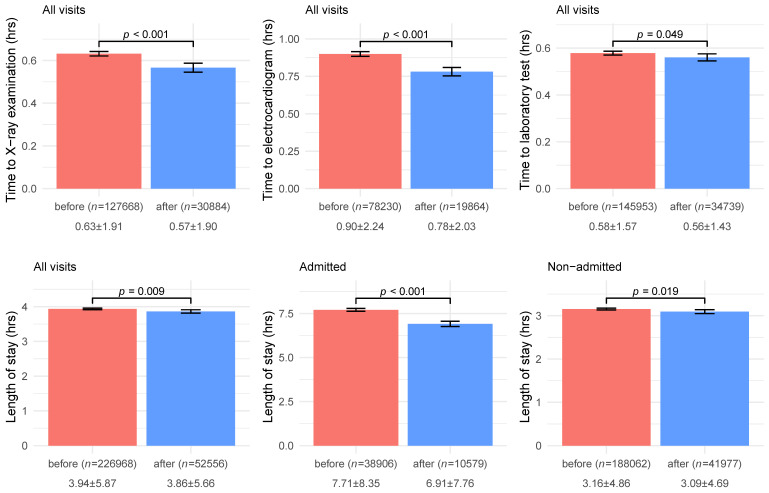
Comparisons of wait time before and after the start of the COVID-19 pandemic. The bar chart and error bar demonstrate the mean and corresponding 95% conference intervals, and the *p*-value is tested by Student’s *t*-test. The annotations below bars are the number of patients and mean ± SD (hours).

Figure 2 shows a comparison of wait time before and after the start of COVID-19. Significantly reduced time to X-ray examination (before: 0.63 ± 1.91 vs. after: 0.57 ± 1.90, *p*-value < 0.001) and time to electrocardiogram (before: 0.90 ± 2.24 vs. after: 0.78 ± 2.03, *p*-value < 0.001) were observed after the start of the COVID-19 pandemic, while a marginally significant decrease was observed in time to laboratory test (before: 0.58 ± 1.57 vs. after: 0.56 ± 1.43, *p*-value = 0.049). The length of stay (before: 3.94 ± 5.87 vs. after: 3.86 ± 5.66, *p*-value = 0.009) also significantly decreased after the start of the COVID-19 pandemic, particularly in admitted visits (before: 7.71 ± 8.35 vs. after: 6.91 ± 7.76, *p*-value < 0.001).

After processing the impact factor analysis of time to examinations, the result shows almost all variables were significantly associated with time to X-ray examination, time to electrocardiogram, and time to laboratory test, except for the association between ED loading parameters and time to laboratory test (data not shown). Table 4 shows that the length of stay was significantly associated with most variables. These potential confounding factors were later adjusted to explore the independent impact of the COVID-19 pandemic. After adjusting for all variables, that time to X-ray significantly decreased, particularly in triage code-level 1, coma, and trauma. The reduction in the time to X-ray and time to electrocardiogram examination after the start of the COVID-19 pandemic might be attributed to the change in ED loading. However, the effect was not significant for time to laboratory test (data not shown). Table 5 shows the change in length of stay after the start of the COVID-19 pandemic. The crude difference of length of stay in admitted visits between before and after the start of the COVID-19 pandemic was −0.80 h (95% CI: −0.97, −0.62), which may not be attributed to the severity of patients [adjusted difference-1: −0.75 (95% CI: −0.93, −0.58)] but rather, to decreased ED loading [adjusted difference-2: −0.02 (95% CI: −0.21, 0.17)]. The major reduction was due to more severe cases, such as triage code-level 1 [difference: −1.58 (95% CI: −2.25, −0.91] and coma [difference: −1.04 (95% CI: −1.73, −0.34]. Admitted visits with fever also benefited from the COVID-19 pandemic, with a reduction in the length of stay of 1.02 h (95% CI: 1.39, 0.66).

**Figure 3:**
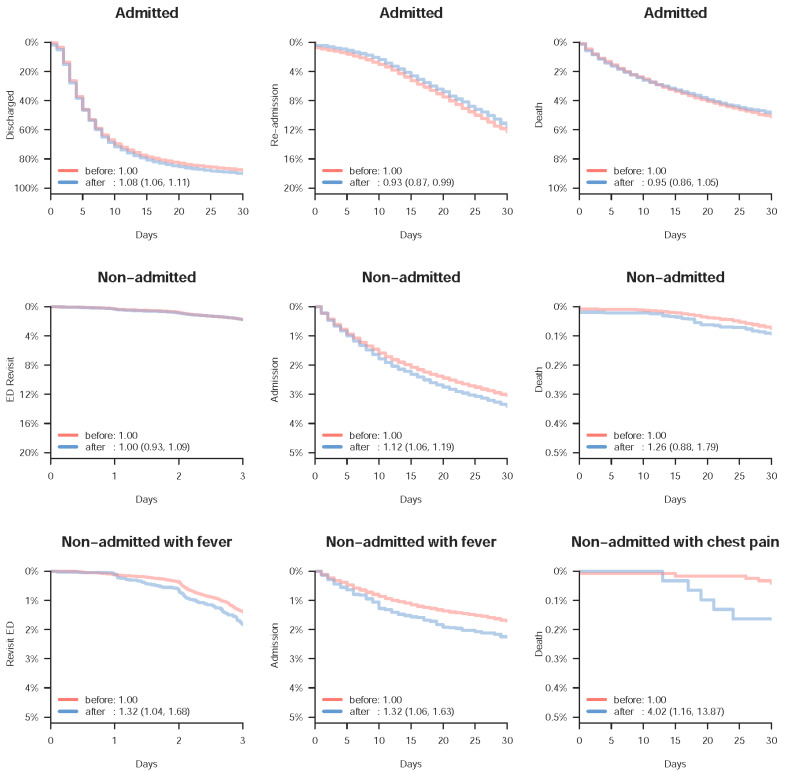
Important prognostic analysis of selected patient groups before and after the start of the COVID-19 pandemic. The Kaplan–Meier curve demonstrates the prognostic difference before (red) and after (blue) the start of the COVID-19 pandemic. Values in the legend are the hazard ratios and corresponding 95% conference intervals based on the Cox proportional hazard model.

Figure 3 shows the change in prognoses after the start of the COVID-19 pandemic. Admitted visits presented with faster discharge without death [HR: 1.08 (95% CI: 1.06, 1.11)] and reduced re-admission [HR: 0.93 (95% CI: 0.87, 0.99)] compared to those before the COVID-19 pandemic. However, there was no difference in mortality among these admitted visits [HR: 0.95 (95% CI: 0.86, 1.05)]. Non-admitted visits exhibited nonsignificant differences in ED revisits [HR: 1.00 (95% CI: 0.93, 1.09)] and mortality [HR: 1.26 (95% CI: 0.88, 1.76)] but significantly more admissions [HR: 1.12 (95% CI: 1.06, 1.19)] were observed. Further stratified analyses revealed that non-admitted visits with fever presented with increased ED revisits [HR: 1.32 (95% CI: 1.04, 1.68)] and admission [HR: 1.32 (95% CI: 1.06, 1.63)]. Of note, non-admitted visits with chest pain presented with a significantly higher mortality [HR: 4.02 (95% CI: 1.16, 13.87)] after the start of the COVID-19 pandemic.

We further analyzed the impact factor of prognoses. Severity indicators, such as triage assessment, physiological state, and disease histories, were significantly associated with worse prognoses, but the effect of ED loading parameters seems not dominant (data not shown). Table 6 shows the change of prognosis after the start of the COVID-19 pandemic in admitted visits. The admitted visits presented significantly faster discharge without death [adjusting HR-2: 1.06 (95% CI: 1.03, 1.08)] and reduced re-admission [adjusting HR-2: 0.92 (95% CI: 0.86, 0.99)]. Table 7 shows the change in prognosis after the start of the COVID-19 pandemic in non-admitted visits. Although non-admitted visits with fever showed a higher risk for ED revisit [crude HR: 1.32 (95% CI: 1.04, 1.68)] and admission [crude HR: 1.12 (95% CI: 1.06, 1.19)], it may be confounded by ED loading [adjusted HR-2: 1.26 (95% CI: 0.96, 1.64) in ED revisit] and baseline characteristics [adjusted HR-1: 1.16 (95% CI: 0.94, 1.44) in admission], respectively. Of note, non-admitted visits with chest pain exhibited increased mortality after the start of the COVID-19 pandemic, which seemed to be an independent effect after adjusting for all variables [adjusting HR-2: 5.40 (95% CI: 1.28, 22.84)]. Therefore, we performed the subgroup analysis in fever and chest pain visits. Visits with fever after the start of COVID-19 presented higher proportions in triage level 2 and comorbidities, both for admitted and non-admitted visits. Although a significant difference in inflammatory markers was present in non-admitted visits (CRP, *p*-value = 0.003; WBC, *p*-value < 0.001), the actual difference may not have clinical importance. Non-admitted visits with chest pain seemed less critical after the start of COVID-19 pandemic, with less proportion in triage level 1 and 2, and less disease comorbidities (data not shown).

## 4. Discussion

ED patient loading significantly decreased during the COVID-19 pandemic studied with a 25% reduction in the number of daily visits. This decrease was attributed to a reduced low acuity presentation and a greater reduction in non-admitted visits. The waiting times to X-ray, electrocardiogram, and laboratory examinations were all reduced due to a significant decrease in ED loading. Moreover, the average length of stay was reduced by 48 min in admitted visits. Admitted visits after the start of the COVID-19 pandemic showed improved prognosis, with significantly faster discharge without death and reduced re-admission. However, non-admitted patients with fever who were subsequently admitted exhibited significantly increased after the start of the COVID-19 pandemic. Importantly, the COVID-19 pandemic independently affects mortality in non-admitted patients with chest pain.

Almost all countries experienced a decrease in medical utilization during the COVID-19 pandemic [13,14,15,16]. This may be attributed to curtailed elective surgery and other noncritical medical services, which are consistence with our data showing a reduction primarily in low acuity non-admitted visits. However, previous studies have also reported puzzling decreases in critical medical demands, such as stroke [29] and acute myocardial infarction [30,31]. The causal relationship between the COVID-19 pandemic and decreased medical utilization remains unclear. Several factors have been hypothesized, such as psychological issues, clinical governance, and social determinants that might change the behaviors of medical service usage [6]. Overall, public fear from COVID-19 pandemic may psychologically deter unnecessary medical utilization and play the major role associated with ED visits reduction in low-risk countries.

The impacts of the COVID-19 pandemic on the ED have primarily manifested as alterations in patient loading and preventive measure implementation. Installation of fever screening stations at EDs to distinguish suspected COVID-19 cases might lead to a shortage of personnel and chaotic traffic flow. Moreover, time to procedures can significantly increase due to preventive measures [11,32]. However, Taiwan showed quick responses and successful technological approaches to mitigate the threats of the COVID-19 pandemic [26,33], which minimized the impact of preventive measures implementation. In contrast, reduced ED loading leads to faster time to examination, as shown in our data illustrating a positive correlation between examination waiting time and ED loading parameters. This study also demonstrated a significantly reduced length of stay in admitted visits; however, the effect was insignificant after adjusting for ED loading parameters, suggesting that a decrease in ED patients predominantly shortens the length of stay. Therefore, the COVID-19 pandemic actually leads to reduced ED loading and faster time to examination in Taiwan, which harbors a low COVID-19 risk.

Fever is the major symptom of COVID-19 cases [34], although three quarters of COVID-19 cases are asymptomatic [35]. Preventive strategies during the COVID-19 pandemic focus on identification and isolation of fever cases with potential contact history facilitated by fever screening stations in health-care systems [9] and imposed self-isolation for individuals [34]. A disproportionate decrease in the length of stay in admitted fever visits represents increased attention and rapid disposition of febrile patients by health-care providers. Moreover, our data demonstrated increased ED revisits and admission in non-admitted visits with fever. The increase might be attributed to the heightened prevention policy and concerns of fever-associated risk from both health-care providers and the public. Moreover, zero death were observed in non-admitted visits with fever after the start of the COVID-19 pandemic, and no difference in the outcome measures of fever cases was observed, both of which may support nationwide efforts for infection prevention and control, such as mandatory mask wearing in public places, limited mass gatherings and social distancing. Accordingly, patients with fever actually benefit from generalized prevention strategies during the COVID-19 pandemic studied.

Non-admitted visits with chest pain demonstrated increased mortality in our study, although these cases were of lower acuity in triage and presented with a reduced proportion of disease history compared to before the COVID-19 pandemic. However, admitted visits with chest pain did not exhibit a significant prognostic difference. Increased mortality in these patients cannot be simply attributed to clinical decisions. A previous study reported patient fear and confusion regarding symptoms are integral parts of this emerging public health crisis, which leads to poor prognosis in ST-elevation myocardial infarction cases [7]. Moreover, there was a 120% increase in OHCA events during the COVID-19 pandemic [5,6]. Since patients with advanced age [36] and more chronic diseases [37,38] have a higher risk for more severe complications from COVID-19, they may be more fearful and hesitant to receive medical care and are more likely to suffer from life-threatening cardiac diseases [39,40]. In addition, past researches also mentioned the increased venous thromboembolism in COVID-19 cases, which might be attributed to delayed intervention during this pandemic [41,42,43]. After the initial COVID-19 pandemic caused significantly decreased hospital admission, the rebound medical demands have not risen to the levels previously observed [25]. The pandemic only caused a remarkably low number of cases of COVID-19 in Taiwan, with a total of 514 patients and only 7 deaths in a population of 24 million during the study period. However, even these low number of cases impacted healthcare, presumably because of changes in behaviors of the health system and the general public. However, authorities should focus on efforts that ensure patients with symptoms of chest pain receive medical care as needed and avoid premature discharge against the advice of the provider.

Certain limitations exist in this study. First, all study samples were from a single medical center; therefore, the results may not be generalizable to hospitals in different settings nationwide. However, no evidence suggests that patient preferences in health-care access decreased after the start of the COVID-19 pandemic. Moreover, this level 1 academic medical center inevitably bears more responsibilities and provides examinations that are more comprehensive during this pandemic. Second, the COVID-19 pandemic may lead to nonemergent surgery or admission being postponed and subsequent increased bed capacity [13,14,15,16]. Therefore, the shorter length of stay in admitted visits may not be due to reduced ED loading entirely. Although health-care service usage involves multifactors, the quality of admitted visits during this pandemic did not decline, but rather, improved. Finally, prognostic analyses lack symptom-onset to ED arrival times. The higher mortality in non-admitted patients with chest pain should be validated in future studies. These delayed visits may become more common after the start of the COVID-19 pandemic and lead to worse prognosis [7]. Nevertheless, our data indicates better outcome at discharge and no difference in 30-day mortality between admitted and non-admitted visits, suggesting the potential delay does not negatively impact the quality of medical care.

The COVID-19 pandemic may cause inevitable public panic, leading to a large reduction in the number of ED visits, particularly in low acuity cases. A reduction in ED visits results in shorter time to examinations and decreased length of stay. The admitted cases benefit from quality improvement with respect to reduced death on discharge and decreased re-admission risk. The change of medical service usage may persist after the COVID-19 vaccine being deployed. However, clinicians should be alert about patients with chest pain due to their increased risk of mortality in subsequent admission. Of note, this experience of a low-risk country suggests that authorities need to educate the public not to neglect life-threatening symptoms, such as chest pain, ensuring that they seek medical service in a timely fashion.

## Figures and Tables

**Table 1:** Comparisons of ED visits before and after the start of COVID-19 pandemic.

	All Visits	Admitted	Non-Admitted
**Crude IRR**			
All	0.745 (0.738–0.752) ⁂	0.875 (0.857–0.894) ⁂	0.718 (0.711–0.726) ⁂
Triage code			
Level 1	0.742 (0.703–0.784) ⁂	0.792 (0.739–0.849) ⁂	0.676 (0.620–0.736) ⁂
Level 2	0.708 (0.691–0.725) ⁂	0.829 (0.797–0.861) ⁂	0.641 (0.621–0.662) ⁂
Level 3	0.751 (0.743–0.759) ⁂	0.912 (0.887–0.939) ⁂	0.728 (0.719–0.736) ⁂
Level 4	0.783 (0.751–0.817) ⁂	1.086 (0.921–1.281)	0.767 (0.735–0.801) ⁂
Disease tag			
Coma	0.844 (0.796–0.895) ⁂	0.890 (0.822–0.965) ⁑	0.795 (0.729–0.867) ⁂
Chest pain	0.818 (0.789–0.849) ⁂	0.934 (0.845–1.033)	0.802 (0.771–0.835) ⁂
Abdomen pain	0.752 (0.732–0.773) ⁂	0.943 (0.891–0.999) *	0.706 (0.685–0.729) ⁂
Trauma	0.783 (0.766–0.800) ⁂	0.930 (0.871–0.993) *	0.768 (0.751–0.786) ⁂
Fever	0.612 (0.596–0.628) ⁂	0.832 (0.796–0.870) ⁂	0.537 (0.520–0.554) ⁂
**Adjusted IRR**			
All	0.749 (0.742–0.756) ⁂	0.875 (0.856–0.894) ⁂	0.723 (0.715–0.730) ⁂
Triage code			
Level 1	0.741 (0.702–0.782) ⁂	0.792 (0.739–0.849) ⁂	0.674 (0.618–0.734) ⁂
Level 2	0.707 (0.690–0.725) ⁂	0.827 (0.796–0.859) ⁂	0.641 (0.621–0.662) ⁂
Level 3	0.755 (0.747–0.763) ⁂	0.912 (0.887–0.938) ⁂	0.732 (0.724–0.741) ⁂
Level 4	0.796 (0.764–0.831) ⁂	1.098 (0.931–1.295)	0.780 (0.747–0.815) ⁂
Disease Tag			
Coma	0.844 (0.796–0.895) ⁂	0.889 (0.821–0.963) ⁑	0.796 (0.730–0.869) ⁂
Chest pain	0.818 (0.788–0.848) ⁂	0.937 (0.847–1.036)	0.801 (0.770–0.834) ⁂
Abdomen pain	0.755 (0.734–0.776) ⁂	0.944 (0.892–1.000) *	0.709 (0.687–0.732) ⁂
Trauma	0.781 (0.764–0.798) ⁂	0.924 (0.865–0.986) *	0.767 (0.749–0.784) ⁂
Fever	0.618 (0.602–0.634) ⁂	0.833 (0.796–0.871) ⁂	0.544 (0.527–0.561) ⁂

The incidence rate ratios (IRR) and corresponding 95% conference intervals were analyzed by Poison regression using daily counts. The adjusted variables included month, holiday, and weekday. * *p* < 0.05; **⁑** *p* < 0.01; **⁂** *p* < 0.001.

**Table 2:** Baseline characteristics before and after the start of the COVID-19 pandemic among admitted and non-admitted visits.

	All Visits (*n* = 279,524)			Admitted (*n* = 49,485)			Non-Admitted (*n* = 230,039)		
	Before (*n* = 226,968)	After (*n* = 52,556)	*p*-Value	Before (*n* = 38,906)	After (*n* = 10,579)	*p*-Value	Before (*n* = 188,062)	After (*n* = 41,977)	*p*-Value
**Triage Assessment**									
Triage code			<0.001			<0.001			<0.001
Level 1	7011 (3.1%)	1617 (3.1%)		4015 (10.3%)	988 (9.3%)		2996 (1.6%)	629 (1.5%)	
Level 2	35,622 (15.7%)	7835 (14.9%)		12,695 (32.6%)	3268 (30.9%)		22,927 (12.2%)	4567 (10.9%)	
Level 3	173,242 (76.3%)	40,404 (76.9%)		21.636 (55.6%)	6134 (58.0%)		15,1606 (80.6%)	34,270 (81.6%)	
Level 4	11,093 (4.9%)	2700 (5.1%)		560 (1.4%)	189 (1.8%)		10,533 (5.6%)	2511 (6.0%)	
Coma	5348 (2.4%)	1403 (2.7%)	<0.001	2758 (7.1%)	763 (7.2%)	0.661	2590 (1.4%)	640 (1.5%)	0.020
Chest pain	13,957 (6.1%)	3548 (6.8%)	<0.001	1695 (4.4%)	492 (4.7%)	0.192	12,262 (6.5%)	3056 (7.3%)	<0.001
Abdomen pain	26,996 (11.9%)	6308 (12.0%)	0.490	5210 (13.4%)	1527 (14.4%)	0.006	21,786 (11.6%)	4781 (11.4%)	0.259
Trauma	43,551 (19.2%)	10,594 (20.2%)	<0.001	3976 (10.2%)	1149 (10.9%)	0.055	39,575 (21.0%)	9445 (22.5%)	<0.001
Fever	36,498 (16.1%)	6937 (13.2%)	<0.001	9272 (23.8%)	2397 (22.7%)	0.012	27,226 (14.5%)	4540 (10.8%)	<0.001
**Physiological State**									
Gender (male)	114,181 (50.3%)	26,500 (50.4%)	0.634	20,143 (51.8%)	5420 (51.2%)	0.324	94,038 (50.0%)	21,080 (50.2%)	0.427
Age (years)	46.8 ± 25.5	49.9 ± 24.3	<0.001	57.5 ± 24.4	58.9 ± 23.1	<0.001	44.6 ± 25.1	47.6 ± 24.0	<0.001
BMI (kg/m^2^)	23.6 ± 82.9	24.2 ± 101.4	0.206	25.5 ± 159.1	25.7 ± 178.1	0.911	23.3 ± 56.7	23.8 ± 71.6	0.097
SBP (mmHg)	133.4 ± 24.7	134.3 ± 24.6	<0.001	134.4 ± 26.5	134.4 ± 26.3	0.906	133.2 ± 24.4	134.2 ± 24.2	<0.001
DBP (mmHg)	76.8 ± 15.9	77.9 ± 15.9	<0.001	77.2 ± 17.5	77.9 ± 17.3	<0.001	76.7 ± 15.6	77.9 ± 15.5	<0.001
Temperature (°C)	36.8 ± 1.0	36.7 ± 0.9	<0.001	37.0 ± 1.1	37.0 ± 1.1	0.002	36.8 ± 0.9	36.7 ± 0.8	<0.001
Pulse (n/min)	88.4 ± 19.6	87.7 ± 19.2	<0.001	92.5 ± 21.1	92.5 ± 20.9	0.947	87.5 ± 19.2	86.5 ± 18.6	<0.001
Breathing (n/min)	18.8 ± 2.5	18.7 ± 2.0	<0.001	19.5 ± 3.2	19.3 ± 2.7	<0.001	18.7 ± 2.3	18.6 ± 1.8	<0.001
**Disease History**									
DM	33,940 (15.0%)	8811 (16.8%)	<0.001	7159 (18.4%)	2096 (19.8%)	0.001	26,781 (14.2%)	6715 (16.0%)	<0.001
HTN	52,761 (23.2%)	13,464 (25.6%)	<0.001	11,462 (29.5%)	3186 (30.1%)	0.190	41,299 (22.0%)	10,278 (24.5%)	<0.001
CKD	16,701 (7.4%)	4373 (8.3%)	<0.001	3610 (9.3%)	1019 (9.6%)	0.268	13,091 (7.0%)	3354 (8.0%)	<0.001
Stroke	23,220 (10.2%)	5437 (10.3%)	0.435	6087 (15.6%)	1478 (14.0%)	<0.001	17,133 (9.1%)	3959 (9.4%)	0.039
CAD	29,346 (12.9%)	7636 (14.5%)	<0.001	5440 (14.0%)	1582 (15.0%)	0.011	23,906 (12.7%)	6054 (14.4%)	<0.001
HLD	44,791 (19.7%)	12,205 (23.2%)	<0.001	6698 (17.2%)	2210 (20.9%)	<0.001	38,093 (20.3%)	9995 (23.8%)	<0.001
**Laboratory Test**									
Cr (mg/dL)	1.3 ± 1.7	1.4 ± 1.8	<0.001	1.3 ± 1.6	1.4 ± 1.7	<0.001	1.3 ± 1.7	1.4 ± 1.8	<0.001
BUN (mg/dL)	22.6 ± 21.1	28.1 ± 26.6	<0.001	25.0 ± 23.4	29.3 ± 27.6	<0.001	21.5 ± 19.9	27.0 ± 25.5	<0.001
AST (U/L)	34.6 ± 100.6	30.8 ± 72.5	<0.001	51.9 ± 149.8	43.8 ± 103.6	<0.001	28.0 ± 73.0	25.3 ± 53.3	<0.001
ALT (U/L)	31.7 ± 92.2	40.4 ± 110.2	<0.001	46.5 ± 143.9	54.1 ± 145.0	0.001	25.2 ± 55.2	28.6 ± 65.2	<0.001
GLU (gm/dL)	137.0 ± 71.0	134.7 ± 69.7	<0.001	149.2 ± 88.1	147.8 ± 86.2	0.157	132.7 ± 63.2	129.3 ± 60.8	<0.001
TnI (pg/mL)	105.9 ± 1568.8	75.9 ± 815.4	0.014	141.7 ± 1481.9	147.6 ± 1195.9	0.781	91.2 ± 1603.2	41.9 ± 545.7	0.001
CRP (mg/L)	2.6 ± 4.9	2.7 ± 5.1	0.515	5.4 ± 7.1	5.4 ± 7.2	0.630	1.7 ± 3.3	1.6 ± 3.4	<0.001
WBC (10^3^/µL)	9.3 ± 5.1	9.1 ± 4.9	<0.001	10.6 ± 6.8	10.5 ± 7.0	0.033	8.8 ± 4.2	8.6 ± 3.6	<0.001
PLT (10^3^/µL)	240.9 ± 86.5	236.3 ± 86.2	<0.001	241.2 ± 104.0	233.8 ± 101.0	<0.001	240.8 ± 79.2	237.4 ± 79.0	<0.001

Abbreviations: BMI, body mass index; SBP, systolic blood pressure; DBP, diastolic blood pressure; DM, diabetes mellitus; HTN, hypertension; CKD, chronic kidney disease; CAD, coronary artery disease; HLD, hyperlipidemia; Cr, creatinine; BUN, blood urea nitrogen; AST, aspartate aminotransferase; ALT, alanine aminotransferase; GLU, glucose AC; TnI, troponin I; CRP, C-reactive protein; WBC, white blood cell count; PLT, platelet.

**Table 3:** ED loading parameters and visit differences by time before and after the start of the COVID-19 pandemic.

	All Visits (*n* = 279,524)			Admitted (*n* = 49,485)			Non-Admitted (*n* = 230,039)		
	Before (*n* = 226,968)	After (*n* = 52,556)	*p*-Value	Before (*n* = 38,906)	After (*n* = 10,579)	*p*-Value	Before (*n* = 188,062)	After (*n* = 41,977)	*p*-Value
**Numbers of Visits at ED**									
Triage code—Level 1	3.5 ± 2.3	2.6 ± 1.8	<0.001	3.6 ± 2.3	2.6 ± 1.8	<0.001	3.5 ± 2.3	2.5 ± 1.8	<0.001
Triage code—Level 2	12.9 ± 5.5	10.0 ± 4.3	<0.001	13.3 ± 5.5	10.1 ± 4.3	<0.001	12.9 ± 5.4	10.0 ± 4.3	<0.001
Triage code—Level 3	34.2 ± 10.0	28.3 ± 8.9	<0.001	33.9 ± 9.7	28.5 ± 8.9	<0.001	34.3 ± 10.0	28.3 ± 8.9	<0.001
Triage code—Level 4	1.2 ± 1.2	1.0 ± 1.1	<0.001	1.1 ± 1.2	1.0 ± 1.1	<0.001	1.2 ± 1.2	1.0 ± 1.2	<0.001
Coma	2.3 ± 1.7	1.8 ± 1.4	<0.001	2.3 ± 1.7	1.8 ± 1.4	<0.001	2.3 ± 1.7	1.8 ± 1.4	<0.001
Chest pain	3.8 ± 2.2	3.0 ± 1.8	<0.001	3.8 ± 2.2	3.0 ± 1.8	<0.001	3.8 ± 2.2	3.0 ± 1.8	<0.001
Abdomen pain	6.7 ± 3.0	4.9 ± 2.5	<0.001	6.6 ± 3.0	5.0 ± 2.5	<0.001	6.7 ± 3.0	4.9 ± 2.5	<0.001
Trauma	6.2 ± 3.1	5.9 ± 3.0	<0.001	6.3 ± 3.2	5.9 ± 3.0	<0.001	6.2 ± 3.1	5.9 ± 3.0	<0.001
Fever	9.7 ± 4.4	6.6 ± 3.4	<0.001	9.5 ± 4.3	6.6 ± 3.4	<0.001	9.7 ± 4.4	6.6 ± 3.4	<0.001
**Time Information**									
Holiday	81,095 (35.7%)	16,649 (31.7%)	<0.001	11,734 (30.2%)	2951 (27.9%)	<0.001	69,361 (36.9%)	13,698 (32.6%)	<0.001
Week			0.004			0.687			0.015
Sunday	38,447 (16.9%)	8536 (16.2%)		5247 (13.5%)	1439 (13.6%)		33,200 (17.7%)	7097 (16.9%)	
Monday	33,090 (14.6%)	7743 (14.7%)		5963 (15.3%)	1617 (15.3%)		27,127 (14.4%)	6126 (14.6%)	
Tuesday	31,058 (13.7%)	7205 (13.7%)		5849 (15.0%)	1550 (14.7%)		25,209 (13.4%)	5655 (13.5%)	
Wednesday	30,482 (13.4%)	7109 (13.5%)		5718 (14.7%)	1522 (14.4%)		24,764 (13.2%)	5587 (13.3%)	
Thursday	29,941 (13.2%)	6981 (13.3%)		5527 (14.2%)	1490 (14.1%)		24,414 (13.0%)	5491 (13.1%)	
Friday	30,526 (13.4%)	7289 (13.9%)		5374 (13.8%)	1527 (14.4%)		25,152 (13.4%)	5762 (13.7%)	
Saturday	33,424 (14.7%)	7693 (14.6%)		5228 (13.4%)	1434 (13.6%)		28,196 (15.0%)	6259 (14.9%)	
**Time Interval**			<0.001			0.990			<0.001
07:30–19:30	135,455 (59.7%)	31,954 (60.8%)		25,908 (66.6%)	7044 (66.6%)		109,547 (58.3%)	24,910 (59.3%)	
19:30–07:30	91,513 (40.3%)	20,602 (39.2%)		12,998 (33.4%)	3535 (33.4%)		78,515 (41.7%)	17,067 (40.7%)	

**Table 4:** Impact factors on time to examination (hours).

	Time to X-ray Examination		Time to Electrocardiogram		Time to Laboratory Test	
	Beta ※	*p*-Value	Beta ※	*p*-Value	Beta ※	*p*-Value
**Triage Assessment**						
Triage code		<0.0001		<0.0001		<0.0001
Level 1	0.00		0.00		0.00	
Level 2	−0.40 (−0.45, −0.35)	<0.0001	0.44 (0.39, 0.50)	<0.0001	0.22 (0.18, 0.25)	<0.0001
Level 3	−0.56 (−0.61, −0.51)	<0.0001	0.78 (0.72, 0.83)	<0.0001	0.27 (0.23, 0.30)	<0.0001
Level 4	−0.63 (−0.71, −0.56)	<0.0001	1.01 (0.85, 1.18)	<0.0001	0.37 (0.31, 0.43)	<0.0001
Coma	0.24 (0.19, 0.30)	<0.0001	−0.28 (−0.34, −0.22)	<0.0001	−0.22 (−0.26, −0.18)	<0.0001
Chest pain	−0.11 (−0.14, −0.07)	<0.0001	−0.76 (−0.80, −0.72)	<0.0001	−0.15 (−0.17, −0.12)	<0.0001
Abdomen pain	0.06 (0.03, 0.08)	0.0001	0.22 (0.18, 0.26)	<0.0001	−0.13 (−0.15, −0.12)	<0.0001
Trauma	−0.27 (−0.29, −0.25)	<0.0001	0.73 (0.67, 0.79)	<0.0001	0.81 (0.78, 0.84)	<0.0001
Fever	0.12 (0.10, 0.15)	<0.0001	0.32 (0.28, 0.36)	<0.0001	0.03 (0.01, 0.05)	0.0006
**Physiological State**						
Gender (male)	−0.10 (−0.12, −0.08)	<0.0001	−0.04 (−0.07, −0.01)	0.0035	−0.03 (−0.05, −0.02)	<0.0001
Age (per 10 years)	0.00 (0.00, 0.01)	0.0496	0.02 (0.01, 0.03)	<0.0001	−0.04 (−0.04, −0.03)	<0.0001
BMI (per 5 kg/m^2^)	0.00 (0.00, 0.00)	<0.0001	−0.01 (−0.03, 0.00)	0.0977	0.00 (0.00, 0.00)	<0.0001
SBP (per 10 mmHg)	−0.02 (−0.02, −0.01)	<0.0001	−0.01 (−0.01, −0.00)	0.0055	−0.00 (−0.00, 0.00)	0.0911
DBP (per 10 mmHg)	−0.02 (−0.02, −0.01)	<0.0001	−0.01 (−0.02, −0.00)	0.0029	0.00 (−0.00, 0.01)	0.6100
Temperature (per 1 °C)	0.05 (0.04, 0.06)	<0.0001	0.12 (0.10, 0.13)	<0.0001	0.03 (0.02, 0.04)	<0.0001
Pulse (per 10/min)	0.03 (0.02, 0.03)	<0.0001	0.01 (0.00, 0.01)	0.0155	−0.00 (−0.01, 0.00)	0.1132
Breathing (per 1/min)	0.02 (0.02, 0.02)	<0.0001	−0.03 (−0.03, −0.02)	<0.0001	0.00 (−0.00, 0.01)	0.0601
**Disease History**						
DM	0.09 (0.06, 0.11)	<0.0001	0.02 (−0.02, 0.05)	0.3202	−0.06 (−0.08, −0.05)	<0.0001
HTN	0.06 (0.04, 0.09)	<0.0001	−0.07 (−0.10, −0.04)	<0.0001	−0.07 (−0.09, −0.06)	<0.0001
CKD	0.13 (0.10, 0.16)	<0.0001	0.05 (0.01, 0.09)	0.0182	−0.05 (−0.08, −0.03)	<0.0001
Stroke	0.14 (0.11, 0.17)	<0.0001	−0.05 (−0.08, −0.01)	0.0108	−0.06 (−0.08, −0.03)	<0.0001
CAD	0.09 (0.06, 0.11)	<0.0001	−0.20 (−0.23, −0.17)	<0.0001	−0.09 (−0.11, −0.07)	<0.0001
HLD	0.04 (0.02, 0.06)	0.0005	−0.04 (−0.07, −0.01)	0.0085	−0.08 (−0.10, −0.06)	<0.0001
**Laboratory Test**						
Cr (per 0.1 mg/dL)	0.00 (−0.00, 0.00)	0.6197	0.00 (0.00, 0.00)	0.0343	−0.00 (−0.00, −0.00)	<0.0001
BUN (per 10 mg/dL)	0.01 (0.01, 0.02)	0.0002	−0.01 (−0.01, −0.00)	0.0274	−0.01 (−0.01, −0.01)	<0.0001
AST (per 20 U/L)	0.01 (0.00, 0.01)	<0.0001	0.00 (−0.00, 0.01)	0.1021	−0.00 (−0.00, 0.00)	0.1304
ALT (per 20 U/L)	0.01 (0.00, 0.01)	0.0003	0.00 (0.00, 0.01)	0.0481	−0.00 (−0.00, −0.00)	0.0001
GLU (per 10 mg/dL)	0.00 (0.00, 0.01)	<0.0001	0.00 (−0.00, 0.00)	0.5529	−0.00 (−0.00, −0.00)	<0.0001
TnI (per 100 pg/mL)	0.00 (0.00, 0.00)	<0.0001	−0.00 (−0.00, 0.00)	0.2880	−0.00 (−0.00, 0.00)	0.1526
CRP (per 1 mg/L)	0.01 (0.00, 0.01)	<0.0001	0.02 (0.02, 0.03)	<0.0001	0.00 (0.00, 0.00)	0.0286
WBC (per 10^3^/µL)	0.01 (0.01, 0.01)	<0.0001	0.01 (0.01, 0.01)	<0.0001	0.00 (0.00, 0.01)	<0.0001
PLT (per 10 × 10^3^/µL)	0.00 (0.00, 0.00)	<0.0001	−0.00 (−0.00, 0.00)	0.0699	0.00 (0.00, 0.00)	<0.0001
**Number of visits**						
Triage code—Level 1	0.01 (0.01, 0.02)	<0.0001	0.03 (0.02, 0.04)	<0.0001	0.00 (−0.00, 0.01)	0.1002
Triage code—Level 2	0.01 (0.00, 0.01)	<0.0001	0.02 (0.02, 0.02)	<0.0001	0.00 (0.00, 0.00)	0.0106
Triage code—Level 3	0.00 (0.00, 0.00)	<0.0001	0.01 (0.01, 0.01)	<0.0001	0.00 (0.00, 0.00)	0.0040
Triage code—Level 4	0.00 (−0.01, 0.01)	0.8606	0.02 (0.01, 0.03)	0.0016	−0.00 (−0.01, 0.01)	0.8722
Trauma	0.01 (0.00, 0.01)	0.0001	0.02 (0.02, 0.03)	<0.0001	0.00 (0.00, 0.01)	<0.0001
Fever	0.01 (0.00, 0.01)	<0.0001	0.02 (0.02, 0.02)	<0.0001	0.00 (0.00, 0.00)	0.0109
Coma	0.02 (0.01, 0.02)	<0.0001	0.04 (0.03, 0.05)	<0.0001	0.00 (0.00, 0.01)	0.0228
Chest pain	0.01 (0.00, 0.01)	0.0003	0.03 (0.02, 0.03)	<0.0001	0.00 (−0.00, 0.00)	0.7525
Abdomen pain	0.01 (0.01, 0.01)	<0.0001	0.02 (0.02, 0.03)	<0.0001	0.00 (0.00, 0.00)	0.0487
**Time Information**						
Holiday	−0.05 (−0.07, −0.03)	<0.0001	−0.08 (−0.10, −0.05)	<0.0001	−0.00 (−0.02, 0.01)	0.6069
Week		0.0012		<0.0001		0.5472
Sunday	0.00		0.00		0.00	
Monday	0.05 (0.01, 0.08)	0.0058	0.07 (0.02, 0.12)	0.0083	0.02 (−0.01, 0.04)	0.2523
Tuesday	0.06 (0.03, 0.10)	0.0005	0.09 (0.03, 0.14)	0.0012	0.01 (−0.02, 0.03)	0.6595
Wednesday	0.05 (0.01, 0.08)	0.0063	0.08 (0.03, 0.13)	0.0024	−0.00 (−0.03, 0.02)	0.8183
Thursday	0.05 (0.01, 0.08)	0.0090	0.07 (0.02, 0.12)	0.0065	0.02 (−0.00, 0.05)	0.0880
Friday	0.03 (−0.00, 0.07)	0.0532	0.03 (−0.03, 0.08)	0.3271	0.01 (−0.02, 0.03)	0.5311
Saturday	0.00 (−0.03, 0.04)	0.8564	−0.03 (−0.09, 0.02)	0.2114	0.01 (−0.02, 0.03)	0.5565
**Time Interval** (19:30—07:30)	0.01 (−0.01, 0.03)	0.4917	−0.13 (−0.16, −0.10)	<0.0001	−0.02 (−0.03, −0.01)	0.0077

※: the beta was presented as estimated value (95% conference intervals) analyzing by linear regression.

**Table 5:** Comparisons of length of stay before and after the start of the COVID-19 pandemic in the emergency department.

	COVID-19 ^€^		Difference ※		
	Before	After	Crude	Adjust (1)	Adjust (2)
**All Visits**					
All	3.94 ± 5.87	3.86 ± 5.66	−0.07 (−0.13, −0.02) ⁑	−0.11 (−0.16, −0.05) ⁂	0.02 (−0.03, 0.08)
Triage Code					
Level 1	9.08 ± 9.34	7.96 ± 8.39	−1.11 (−1.61, −0.62) ⁂	−1.14 (−1.63, −0.65) ⁂	−0.22 (−0.76, 0.31)
Level 2	6.37 ± 7.12	6.21 ± 6.78	−0.16 (−0.33, 0.01)	−0.25 (−0.42, −0.07) ⁑	0.10 (−0.09, 0.29)
Level 3	3.36 ± 5.21	3.38 ± 5.13	0.01 (−0.04, 0.07)	−0.05 (−0.10, 0.01)	0.01 (−0.05, 0.07)
Level 4	1.80 ± 4.28	1.88 ± 4.51	0.08 (−0.10, 0.26)	0.07 (−0.11, 0.25)	0.05 (−0.15, 0.25)
Disease Tag					
Coma	7.81 ± 8.18	7.10 ± 7.58	−0.71 (−1.18, −0.23) ⁑	−0.67 (−1.14, −0.20) ⁑	0.13 (−0.38, 0.64)
Chest pain	4.83 ± 6.43	4.62 ± 5.94	−0.21 (−0.45, 0.02)	−0.10 (−0.32, 0.12)	0.11 (−0.13, 0.35)
Abdomen pain	4.43 ± 5.50	4.33 ± 5.53	−0.10 (−0.25, 0.05)	−0.17 (−0.32, −0.03) *	−0.04 (−0.20, 0.12)
Trauma	2.39 ± 4.25	2.42 ± 4.52	0.03 (−0.06, 0.12)	−0.00 (−0.09, 0.09)	−0.03 (−0.13, 0.06)
Fever	4.56 ± 6.54	4.64 ± 6.11	0.08 (−0.09, 0.25)	−0.25 (−0.42, −0.09) ⁑	0.00 (−0.17, 0.18)
**Admitted**					
All	7.71 ± 8.35	6.91 ± 7.76	−0.80 (−0.97, −0.62) ⁂	−0.75 (−0.93, −0.58) ⁂	−0.02 (−0.21, 0.17)
Triage Code					
Level 1	9.85 ± 9.86	8.27 ± 8.56	−1.58 (−2.25, −0.91) ⁂	−1.73 (−2.40, −1.07) ⁂	−0.57 (−1.29, 0.15)
Level 2	8.06 ± 8.25	7.26 ± 7.76	−0.79 (−1.10, −0.48) ⁂	−0.82 (−1.13, −0.50) ⁂	−0.06 (−0.40, 0.28)
Level 3	7.11 ± 7.97	6.52 ± 7.52	−0.59 (−0.82, −0.37) ⁂	−0.57 (−0.79, −0.35) ⁂	0.06 (−0.18, 0.31)
Level 4	7.55 ± 10.06	6.42 ± 9.72	−1.13 (−2.77, 0.52)	−1.32 (−2.91, 0.26)	−0.89 (−2.72, 0.94)
Disease Tag					
Coma	8.78 ± 8.80	7.74 ± 8.11	−1.04 (−1.73, −0.34) ⁑	−1.03 (−1.72, −0.34) ⁑	0.22 (−0.52, 0.97)
Chest pain	9.65 ± 9.87	8.83 ± 9.46	−0.82 (−1.80, 0.17)	−0.77 (−1.73, 0.19)	0.42 (−0.63, 1.46)
Abdomen pain	7.68 ± 7.54	6.80 ± 7.07	−0.89 (−1.31, −0.46) ⁂	−0.87 (−1.29, −0.44) ⁂	−0.40 (−0.86, 0.06)
Trauma	6.63 ± 7.28	6.13 ± 7.42	−0.50 (−0.98, −0.02) *	−0.42 (−0.89, 0.05)	0.08 (−0.44, 0.60)
Fever	7.48 ± 8.37	6.46 ± 7.27	−1.02 (−1.39, −0.66) ⁂	−1.01 (−1.38, −0.64) ⁂	−0.14 (−0.54, 0.25)
**Non-Admitted**					
All	3.16 ± 4.86	3.09 ± 4.69	−0.06 (−0.11, −0.01) *	−0.08 (−0.13, −0.03) ⁑	−0.06 (−0.11, −0.00) *
Triage Code					
Level 1	8.04 ± 8.49	7.48 ± 8.09	−0.56 (−1.28, 0.17)	−0.56 (−1.26, 0.15)	0.05 (−0.71, 0.82)
Level 2	5.44 ± 6.21	5.46 ± 5.88	0.02 (−0.18, 0.21)	−0.10 (−0.30, 0.09)	0.01 (−0.20, 0.22)
Level 3	2.83 ± 4.43	2.81 ± 4.34	−0.02 (−0.07, 0.04)	−0.07 (−0.12, −0.02) ⁑	−0.08 (−0.13, −0.02) ⁑
Level 4	1.49 ± 3.48	1.54 ± 3.62	0.04 (−0.11, 0.20)	0.03 (−0.12, 0.18)	0.00 (−0.16, 0.17)
Disease Tag					
Coma	6.77 ± 7.31	6.34 ± 6.82	−0.44 (−1.06, 0.19)	−0.49 (−1.10, 0.12)	−0.10 (−0.77, 0.57)
Chest pain	4.17 ± 5.48	3.94 ± 4.82	−0.22 (−0.44, −0.01) *	−0.11 (−0.31, 0.10)	−0.06 (−0.29, 0.16)
Abdomen pain	3.65 ± 4.56	3.55 ± 4.68	−0.11 (−0.25, 0.04)	−0.18 (−0.32, −0.04) *	−0.13 (−0.28, 0.02)
Trauma	1.97 ± 3.55	1.97 ± 3.79	0.00 (−0.08, 0.08)	−0.02 (−0.09, 0.06)	−0.09 (−0.18, −0.01) *
Fever	3.56 ± 5.44	3.68 ± 5.15	0.11 (−0.06, 0.28)	−0.11 (−0.28, 0.05)	−0.04 (−0.22, 0.14)

^€^ the length of stay was presented as the mean ± SD (hours); ※ the difference was presented as the mean (95% conference intervals); the adjust (1) variables included triage assessment (triage code, coma, chest pain, abdomen pain, trauma, and fever), physiological state (gender, age, BMI, SBP, DBP, temperature, pulse, and breathing), and disease history (DM, HTN, CKD, Stroke, CAD, and HLD); the adjust (2) variables additionally included ED loading parameters (number of all subgroups of triage assessment, date information, and time information). * *p* < 0.05; **⁑** *p* < 0.01; **⁂** *p* < 0.001.

**Table 6:** Prognosis of admitted visits before and after the start of the COVID-19 pandemic.

	COVID-19 ^€^		Hazard Ratio ※		
	Before	After	Crude	Adjust (1)	Adjust (2)
**Discharge without Death ^†^**					
All	36,728 (94.40%)	10,059 (95.08%)	1.08 (1.06, 1.11) ⁂	1.08 (1.05, 1.10) ⁂	1.06 (1.03, 1.08) ⁂
Triage code					
Level 1	3122 (77.76%)	758 (76.72%)	0.97 (0.89, 1.05)	0.99 (0.91, 1.07)	1.00 (0.91, 1.09)
Level 2	11,939 (94.04%)	3108 (95.10%)	1.05 (1.01, 1.09) *	1.06 (1.02, 1.10) ⁑	1.05 (1.00, 1.10) *
Level 3	21,125 (97.64%)	6006 (97.91%)	1.08 (1.05, 1.11) ⁂	1.07 (1.04, 1.10) ⁂	1.05 (1.01, 1.08) ⁑
Level 4	542 (96.79%)	187 (98.94%)	1.55 (1.30, 1.83) ⁂	1.40 (1.18, 1.67) ⁂	1.48 (1.21, 1.80) ⁂
Disease tag					
Coma	2287 (82.92%)	650 (85.19%)	1.15 (1.05, 1.25) ⁑	1.15 (1.05, 1.25) ⁑	1.17 (1.06, 1.29) ⁑
Chest pain	1621 (95.63%)	469 (95.33%)	1.04 (0.94, 1.15)	1.06 (0.96, 1.18)	1.06 (0.95, 1.20)
Abdomen pain	5021 (96.37%)	1485 (97.25%)	1.09 (1.03, 1.16) ⁑	1.06 (1.00, 1.12) *	1.00 (0.94, 1.07)
Trauma	3875 (97.46%)	1130 (98.35%)	1.22 (1.14, 1.30) ⁂	1.18 (1.10, 1.26) ⁂	1.16 (1.08, 1.25) ⁂
Fever	8856 (95.51%)	2306 (96.20%)	1.02 (0.97, 1.07)	1.04 (0.99, 1.09)	1.04 (0.99, 1.10)
**Re-Admission in 30 Days**					
All	4503 (12.26%)	1155 (11.48%)	0.93 (0.87, 0.99) *	0.91 (0.86, 0.97) ⁑	0.92 (0.86, 0.99) *
Triage code					
Level 1	385 (12.33%)	91 (12.01%)	0.97 (0.77, 1.22)	0.94 (0.74, 1.18)	0.93 (0.73, 1.20)
Level 2	1668 (13.97%)	419 (13.48%)	0.96 (0.86, 1.06)	0.93 (0.84, 1.04)	0.93 (0.82, 1.04)
Level 3	2377 (11.25%)	634 (10.56%)	0.93 (0.85, 1.02)	0.91 (0.84, 1.00) *	0.92 (0.84, 1.02)
Level 4	73 (13.47%)	11 (5.88%)	0.42 (0.22, 0.80) ⁑	0.45 (0.23, 0.86) *	0.54 (0.26, 1.10)
Disease tag					
Coma	326 (14.25%)	88 (13.54%)	0.93 (0.74, 1.18)	0.92 (0.72, 1.16)	0.90 (0.70, 1.16)
Chest pain	197 (12.15%)	47 (10.02%)	0.82 (0.59, 1.12)	0.77 (0.56, 1.06)	0.77 (0.54, 1.10)
Abdomen pain	645 (12.85%)	166 (11.18%)	0.86 (0.73, 1.02)	0.88 (0.74, 1.04)	0.84 (0.70, 1.02)
Trauma	233 (6.01%)	68 (6.02%)	1.00 (0.76, 1.31)	0.97 (0.74, 1.28)	0.98 (0.72, 1.32)
Fever	1085 (12.25%)	280 (12.14%)	0.99 (0.86, 1.12)	0.92 (0.80, 1.05)	0.90 (0.78, 1.03)
**Death in 30 days**					
All	1989 (5.11%)	514 (4.86%)	0.95 (0.86, 1.05)	0.96 (0.87, 1.06)	0.97 (0.87, 1.08)
Triage code					
Level 1	794 (19.78%)	212 (21.46%)	1.10 (0.95, 1.28)	1.05 (0.90, 1.22)	1.05 (0.89, 1.24)
Level 2	724 (5.70%)	177 (5.42%)	0.95 (0.81, 1.12)	0.91 (0.78, 1.08)	0.96 (0.80, 1.15)
Level 3	456 (2.11%)	124 (2.02%)	0.96 (0.79, 1.17)	0.94 (0.77, 1.15)	0.93 (0.74, 1.15)
Level 4	15 (2.68%)	1 (0.53%)	0.20 (0.03, 1.48)	0.27 (0.03, 2.22)	0.17 (0.02, 1.76)
Disease tag					
Coma	422 (15.30%)	99 (12.98%)	0.84 (0.67, 1.04)	0.81 (0.65, 1.01)	0.78 (0.61, 0.99) *
Chest pain	62 (3.66%)	21 (4.27%)	1.17 (0.71, 1.91)	1.10 (0.66, 1.83)	0.99 (0.55, 1.78)
Abdomen pain	174 (3.34%)	38 (2.49%)	0.74 (0.52, 1.05)	0.83 (0.58, 1.18)	0.94 (0.64, 1.38)
Trauma	89 (2.24%)	19 (1.65%)	0.74 (0.45, 1.21)	0.80 (0.49, 1.32)	0.90 (0.52, 1.55)
Fever	362 (3.90%)	94 (3.92%)	1.01 (0.80, 1.26)	0.99 (0.79, 1.24)	1.08 (0.84, 1.37)

^€^ the data were presented as number of events (proportion); ^†^ higher HR means better; ※ the difference was presented as hazard ratio (95% conference intervals); the adjust (1) variables included triage assessment (triage code, coma, chest pain, abdomen pain, trauma, and fever), physiological state (gender, age, BMI, SBP, DBP, temperature, pulse, and breathing), and disease history (DM, HTN, CKD, Stroke, CAD, and HLD); the adjust (2) variables additionally included ED loading parameters (number of all subgroups of triage assessment, date information, and time information). * *p* < 0.05; **⁑** *p* < 0.01; **⁂** *p* < 0.001.

**Table 7:** Prognosis of non-admitted visits before and after the start of the COVID-19 pandemic.

	COVID-19 Pandemic ^€^		Hazard Ratio ※		
	Before	After	Crude	Adjust (1)	Adjust (2)
**ED revisit in 3 Days**					
All	3313 (1.76%)	742 (1.77%)	1.00 (0.93, 1.09)	0.96 (0.88, 1.03)	0.96 (0.88, 1.05)
Triage code					
Level 1	27 (0.90%)	5 (0.79%)	0.88 (0.34, 2.29)	0.90 (0.34, 2.34)	0.63 (0.20, 1.98)
Level 2	409 (1.78%)	91 (1.99%)	1.12 (0.89, 1.40)	1.09 (0.87, 1.37)	1.09 (0.85, 1.40)
Level 3	2507 (1.65%)	575 (1.68%)	1.02 (0.93, 1.11)	0.96 (0.88, 1.05)	0.97 (0.88, 1.07)
Level 4	370 (3.51%)	71 (2.83%)	0.80 (0.62, 1.03)	0.90 (0.70, 1.16)	0.90 (0.68, 1.20)
Disease tag					
Coma	34 (1.31%)	12 (1.88%)	1.43 (0.74, 2.77)	1.35 (0.69, 2.62)	1.37 (0.65, 2.93)
Chest pain	249 (2.03%)	48 (1.57%)	0.77 (0.57, 1.05)	0.78 (0.57, 1.06)	0.86 (0.62, 1.20)
Abdomen pain	319 (1.46%)	65 (1.36%)	0.93 (0.71, 1.21)	0.86 (0.65, 1.12)	0.91 (0.68, 1.21)
Trauma	427 (1.08%)	89 (0.94%)	0.87 (0.69, 1.10)	0.83 (0.66, 1.04)	0.76 (0.59, 0.98) *
Fever	377 (1.38%)	83 (1.83%)	1.32 (1.04, 1.68) *	1.33 (1.05, 1.69) *	1.26 (0.96, 1.64)
**Admission in 30 Days**					
All	5718 (3.04%)	1431 (3.41%)	1.12 (1.06, 1.19) ⁂	1.07 (1.01, 1.14) *	1.09 (1.03, 1.16) ⁑
Triage code					
Level 1	81 (2.71%)	15 (2.39%)	0.88 (0.51, 1.53)	1.00 (0.58, 1.75)	1.25 (0.69, 2.28)
Level 2	985 (4.30%)	239 (5.24%)	1.23 (1.06, 1.41) ⁑	1.22 (1.06, 1.41) ⁑	1.26 (1.08, 1.47) ⁑
Level 3	4317 (2.85%)	1101 (3.21%)	1.13 (1.06, 1.21) ⁂	1.06 (0.99, 1.13)	1.07 (1.00, 1.15)
Level 4	335 (3.18%)	76 (3.03%)	0.95 (0.74, 1.22)	0.98 (0.76, 1.25)	1.03 (0.78, 1.35)
Disease tag					
Coma	81 (3.13%)	21 (3.28%)	1.05 (0.65, 1.69)	1.08 (0.66, 1.75)	1.52 (0.90, 2.56)
Chest pain	340 (2.77%)	103 (3.37%)	1.22 (0.98, 1.52)	1.28 (1.03, 1.60) *	1.59 (1.25, 2.03) ⁂
Abdomen pain	768 (3.53%)	207 (4.33%)	1.24 (1.06, 1.44) ⁑	1.14 (0.97, 1.33)	1.10 (0.93, 1.31)
Trauma	712 (1.80%)	157 (1.66%)	0.92 (0.78, 1.10)	0.88 (0.74, 1.05)	0.88 (0.73, 1.07)
Fever	466 (1.71%)	102 (2.25%)	1.32 (1.06, 1.63) *	1.16 (0.94, 1.44)	1.10 (0.87, 1.39)
**Death in 30 Days**					
All	139 (0.07%)	39 (0.09%)	1.26 (0.88, 1.79)	1.18 (0.83, 1.69)	1.23 (0.83, 1.80)
Triage code					
Level 1	5 (0.17%)	3 (0.48%)	2.86 (0.68, 11.97)	3.52 (0.78, 15.81)	1.94 (0.32, 11.72)
Level 2	26 (0.11%)	8 (0.18%)	1.55 (0.70, 3.41)	1.49 (0.68, 3.30)	1.84 (0.76, 4.41)
Level 3	100 (0.07%)	25 (0.07%)	1.11 (0.71, 1.71)	1.00 (0.64, 1.55)	1.02 (0.63, 1.63)
Level 4	8 (0.08%)	3 (0.12%)	1.57 (0.42, 5.93)	1.22 (0.27, 5.47)	1.75 (0.32, 9.53)
Disease tag					
Coma	6 (0.23%)	0 (0.00%)	0.00 (0.00, Inf)	0.00 (0.00, Inf)	0.00 (0.00, Inf)
Chest pain	5 (0.04%)	5 (0.16%)	4.02 (1.16, 13.87) *	5.01 (1.40, 17.86) *	5.40 (1.28, 22.84) *
Abdomen pain	23 (0.11%)	5 (0.10%)	0.99 (0.38, 2.61)	0.77 (0.29, 2.04)	0.79 (0.28, 2.25)
Trauma	10 (0.03%)	5 (0.05%)	2.10 (0.72, 6.13)	2.07 (0.70, 6.09)	2.50 (0.72, 8.69)
Fever	5 (0.02%)	0 (0.00%)	0.00 (0.00, Inf)	0.00 (0.00, Inf)	0.00 (0.00, Inf)

^€^ the data were presented as number of events (proportion); ※: the difference was presented as hazard ratio (95% conference intervals); the adjust (1) variables included triage assessment (triage code, coma, chest pain, abdomen pain, trauma, and fever), physiological state (gender, age, BMI, SBP, DBP, temperature, pulse, and breathing), and disease history (DM, HTN, CKD, Stroke, CAD, and HLD); the adjust (2) variables additionally included ED loading parameters (number of all subgroups of triage assessment, date information, and time information). * *p* < 0.05; **⁑** *p* < 0.01; **⁂** *p* < 0.001.

## Data Availability

The data presented in this study are available on request from the corresponding author.
